# Comprehension of Mandarin Aspect Markers by Preschool Children With and Without Developmental Language Disorder

**DOI:** 10.3389/fpsyg.2022.839951

**Published:** 2022-04-28

**Authors:** Lijun Chen, Stephanie Durrleman

**Affiliations:** ^1^Center for Linguistics and Applied Linguistics, Guangdong University of Foreign Studies, Guangzhou, China; ^2^ABCCD – Autism, Bilingualism, Cognitive and Communicative Development Lab, Faculty of Science and Medicine, University of Fribourg, Fribourg, Switzerland

**Keywords:** developmental language disorder, preschool children, Mandarin Chinese, aspect markers, comprehension, delayed

## Abstract

Children with developmental language disorder (DLD) reportedly struggle with the comprehension of aspect. However, since aspect and tense are closely entangled in the languages spoken by the children with DLD in previous studies, it is unclear whether the difficulty stems from aspect, tense, or both. Mandarin Chinese, a language without morphological manifestations of tense, is ideal to investigate whether the comprehension of aspect is specifically affected in children with DLD, yet to date work on this is scarce and presents methodological limitations. In this study, we examined whether preschool Mandarin-speaking children with DLD have difficulty in comprehending perfective aspect (represented with the aspect marker -*le*) compared to imperfective aspect (represented with the aspect markers *zai-* and -*zhe*), whether performance can be explained in terms of the pre- vs. post-verbal realization of the aspect markers, and the potential role played by lexical aspect in the comprehension of grammatical aspect. Fourteen preschool children with DLD (mean age: 61.11 months old) and 14 TD children (mean age: 63.4 months old) matched for age and nonverbal intelligence participated in a sentence-picture matching task. Global results showed that, similar to their TD peers, children with DLD performed better on imperfective aspect than perfective aspect. Concerning specific aspect markers, while children with DLD indeed performed similarly to TD children on imperfective *-zhe*, they obtained significantly lower accuracy than TD children on perfective *-le* and imperfective *zai-*. However, considering verb types combined with these aspect markers, results revealed that children with DLD scored significantly higher on the prototypical combination(s) (e.g., *zai-* + Activity verbs) than on the non-prototypical combination(s) (e.g., *zai-* + Accomplishment verbs). The performance pattern suggests that the comprehension of aspect markers by children with DLD is particularly affected by lexical aspect. As this also affects younger TD children, children with DLD are arguably at an earlier stage of aspectual development than their age and nonverbal intelligence matched TD peers. Therefore, the aspectual development of children with DLD appears to be delayed rather than deviant. Given this, language programs addressing difficulties in DLD may need to incorporate training on the use of aspect markers, especially targeting their combination with non-prototypical verbs.

## Introduction

Developmental Language Disorder (hereafter, DLD), also previously called Specific Language Impairment, refers to a language impairment that does not stem from a known biomedical etiology (Bishop et al., 2017). Children with DLD have deficits in grammatical abilities, in particular tense (Wexler et al., 2004; Bishop, 2014; Krok and Leonard, 2015; Chondrogianni and John, 2018; Deevy and Leonard, 2018). Therefore, when acquiring a language such as English, children with DLD tend to omit grammatical inflections (such as *-ed* and *-s*) and use nonfinite forms of lexical verbs (i.e., bare verb forms) more frequently than typically developing (hereafter, TD) children. Intervention has proven beneficial for addressing grammatical deficits of children with DLD (Leonard et al., 2006; Garraffa et al., 2018; Calder et al., 2021), with the careful selection of treatment stimuli playing a crucial role in outcomes (Owen Van Horne et al., 2018). In contrast to the vast amount of studies on the challenges with tense displayed by children with DLD, much less attention has been given to potential difficulties with aspect, which is the focus of the current work.

Aspect concerns the internal temporal structure of situations introduced into a discourse (Smith, 1994). The domain of aspect includes temporal viewpoints and temporal situation types (Smith, 1994, 1997; Smith and Erbaugh, 2005). The temporal situation types, also called lexical aspect or situation aspect, are realized by the predicate verb and its object(s), the “verb constellation.” Verb constellations that are associated with a given situation type have unique distributional and semantic properties (Smith and Erbaugh, 2005). Using three two-valued temporal features, [±dynamic], [±durative] and [±telic], Vendler (1967) differentiates the main classes of English verb constellations into four types, including State, Activity, Accomplishment, and Achievement. Temporal viewpoint, also called grammatical aspect, conveys a temporal perspective that focuses on all or part of that situation (Smith, 1994). Commonly, there are two types of temporal viewpoints, namely, imperfective viewpoint (or imperfective aspect) and perfective viewpoint (or perfective aspect). Imperfective aspect focuses on intervals that are neither initial nor final, thus excluding endpoints, while perfective aspect includes both endpoints of a situation and are closed informationally (Smith, 1994).

Lexical aspect has a strong impact on L1 mastery of grammatical aspect, in both children and adults (Li and Shirai, 2000; Shirai, 2011). For example, four- to six-year-old Mandarin-speaking children and 18-year-old Cantonese-speaking adults fare better when aspect markers are combined with their prototypical verbs (e.g., the perfective markers with Accomplishment verbs and the imperfective markers with Activity verbs) than with non-prototypical verbs (perfective markers with Activity verbs and imperfective markers with Accomplishment verbs) in a forced sentence-picture matching task (Li and Bowerman, 1998; Yap et al., 2009). The interaction between lexical aspect and grammatical aspect is also revealed in L2 contexts (Bardovi-Harlig and Comajoan-Colomé, 2020). For instance, 20-year-old native Mandarin speakers learning English process sentences faster when regular tense-aspect markers (-*ed* and *-ing*) are combined with the prototypical verbs than with non-prototypical verbs in a self-paced reading task, and they spend a longer time than native English speakers only when these markers are combined with the non-prototypical verbs (Zeng et al., 2021). These effects of lexical aspect on grammatical aspect are summarized by the Aspect Hypothesis (Andersen and Shirai, 1994, 1996). Namely, language learners combine the past or perfective markers with Achievement or Accomplishment verbs first and then extend to Activity and State verbs, while they combine the progressive markers first with Activity verbs and then extend to Accomplishment and Achievement verbs. Finally, imperfective past emerges later than perfective past in languages that encode the perfective-imperfective distinction. Shirai and Andersen (1995) found that children only combine both regular and irregular past tense morphology with Achievement verbs at first and then extend to Accomplishment, Activity, and State verbs. Similar findings were reported in Turkish-speaking children who first use the past inflection with Achievement verbs (Aksu-Koc, 1998) and in Mandarin-speaking children who combine the perfective marker -*le* with Achievement verbs first, and then move to Accomplishment and Activity verbs (Jin and Hendriks, 2005; Chen and Shirai, 2010).

It is controversial whether lexical aspect impacts the use of grammatical aspect by children with DLD. English-speaking children with DLD do not show the effect of lexical aspect on the production of grammatical aspect (Leonard et al., 2007) nor on the comprehension of grammatical aspect (Leonard and Deevy, 2010; Stuart and van der Lely, 2015). Studies on Spanish-speaking children with DLD exhibit the effect of lexical aspect on the production of grammatical aspect (Grinstead et al., 2016), but not on the comprehension of grammatical aspect (Grinstead et al., 2013). Unlike English- and Spanish-speaking children with DLD, children with DLD who speak Chinese languages perform better when the aspect markers are combined with their prototypical verbs than with non-prototypical verbs in production tasks (Stokes and Fletcher, 2003; Cheung, 2005; He et al., 2013; Chen et al., 2021) as well as in comprehension tasks (Yu et al., 2019).

Focusing on the comprehension of perfective aspect and imperfective aspect, English-speaking (Stuart and van der Lely, 2015) and Greek-speaking (Konstantzou, 2015; Dosi, 2019) children and adolescents with DLD, like language-matched TD children, perform better on comprehending perfective aspect than on imperfective aspect under past tense. On the contrary, Turkish-speaking children with DLD perform better on comprehending imperfective aspect than on perfective aspect under present tense (Duman and Topbas, 2016). These controversial findings could be ascribed to the interaction between tense and aspect. Specifically, perfective aspect and the past tense belong to naturally conflated linguistic categories, as well as imperfective aspect and the present tense (Wagner, 2001). In English, for instance, both past tense and perfective aspect cue the completion of events; in contrast, progressive aspect (one form of imperfective aspect) and present tense cue events that are “here and now.” Since comprehending the imperfective past requires combining the “ongoingness” marked by *-ing* with the “pastness” marked by the past tense, the semantic operation for imperfective past is more complex than that for perfective aspect. Put differently, “capturing completed events unambiguously is easier than ensuring that the ongoingness of events that have taken place in the past” (Stuart and van der Lely, 2015: 198). Following van Hout’s Semantic Complexity Hypothesis whereby the semantics of simple semantic operations is acquired early (2008: 1753), we would expect imperfective to emerge later than perfective when these occur in combination with past tense. Therefore, the interaction between tense and aspect may explain why young children acquire imperfective past later than perfective past and perform worse on imperfective past than on perfective past. In contrast, comprehending the imperfective aspect in the present tense might be easier than in the past tense, which is indeed confirmed by the findings of Leonard and Deevy (2010) that children with DLD performed better on comprehending present progressive events than past progressive events. In this study, we aim to further explore the asymmetry between imperfective aspect and perfective aspect and provide empirical data from a tense-less language: Mandarin Chinese (hereafter, Mandarin). Mandarin is an ideal language to examine aspect solely since it is an aspect-prominent and tense-less language that has no overt grammatical inflections or auxiliaries appearing in the sentence to express tense^1^ (Smith, 1994; Li and Bowerman, 1998; Li and Shirai, 2000; Lin, 2003; Li, 2012). However, no study thus far has compared the comprehension of perfective aspect and imperfective aspect by Mandarin-speaking children with DLD.

### Aspect in Mandarin

Mandarin has stricter word order than English, with over 90% of Mandarin sentences adhering to the structure “Subject + verb + object” (Erbaugh, 1992). Mandarin temporality is presented by grammatical aspect, lexical aspect, adverbs, and pragmatic principles (Smith and Erbaugh, 2005). Grammatical aspect in Mandarin is expressed by overt markers (Smith, 1994). Imperfective aspect is expressed by the aspect markers *zai-* and *-zhe*. The pre-verbal *zai-* is pronounced with the falling tone and is used to indicate the ongoing or progressive stage of an event (Chen, 1999; Xiao and McEnery, 2004), as shown in (1). The post-verbal *-zhe* is pronounced without stress and with the neutral tone in a sentence. It is regarded as a durative marker that indicates the duration of a state or event (Li and Thompson, 1981; Zhu, 1982). *-zhe* expresses a resultative state^2^ rather than the subinterval of a dynamic event (Smith, 1997; Jin and Hendriks, 2005), as illustrated in (2).

(1) John   **zai**    xi   yifu.
  ZAI wash clothes.
  “John is washing clothes.”
(2)  Wangshushu chuan-**zhe** yi-jian maoyi.
  Uncle Wang wear-ZHE a-CL  sweater.
  “Uncle Wang is wearing a sweater.”

Being pronounced with the neutral tone and without stress in a sentence, *-le* expresses perfective aspect in Mandarin when it follows the predicate verb. It indicates the completion or arbitrary termination of an event (Chao, 1968; Lin, 2003), as shown in (3).

(3) Zhangsan jian-**le** yi-zuo fangzi.
  Zhangsan build-LE a-CL house.
  “Zhangsan built a house.”

Thirty-month-old Mandarin-speaking TD children are sensitive to the aspectual contrast between the perfective marker -*le* and the imperfective marker *-zhe* in a preferential looking experiment (Yang et al., 2018). As they approach 3 years of age, TD children can utilize the cues of the temporal information encoded in the aspect markers *-l*e and *-zhe* to recognize events as well as adults, as indicated by their eyes moving to aspect marker-matched ongoing or completed event areas during an eye-tracking experiment (Zhou et al., 2014). TD children also have already mastered the functions of aspect markers *zai-*, *-le*, and *-zhe* in discourse and do not overuse or underuse aspect markers in their production when they are 7 years old (Jin and Hendriks, 2005). Erbaugh (1992) revealed that the perfective marker *-le* emerges earliest, then the progressive marker *zai-* and the durative marker -*zhe*, and finally the experiential marker *-guo* in TD children’s production.

The acquisition of Mandarin aspect markers by TD children is impacted by lexical aspect. There are five types of verbs in Mandarin, including Semelfactive [(+dynamic) (-durative)(-telic); e.g., *ti* “kick” and *kesu* “cough”], State [(-dynamic)(+durative)(-telic); e.g., *xihuan* “like” and *zhidao* “know”], Activity [(+dynamic) (+durative)(-telic); e.g., *pao* “run” and *chi* “eat”], Accomplishment [(+dynamic) (+durative) (+telic); e.g., *hua yifu hua* “draw a picture” and *gai yizuo fangzi* “build a house”], and Achievement [(+dynamic)(-durative)(+telic); e.g., *si* “die” and *dapo* “break”; Smith, 1994, 1997]. The impact of lexical aspect on the acquisition of grammatical aspect is consistent with the Aspect Hypothesis. Namely, TD children tend to combine the perfective marker *-le* with Achievement verbs, the progressive marker *zai-* with Activity verbs, and the durative marker *-zhe* with Activity and State verbs (Jin and Hendriks, 2005; Chen and Shirai, 2010), although this tendency weakens over time. In this study, we consider the verbs that are combined firstly with each aspect marker as its prototypical verbs, and those that are combined later with each aspect marker as its non-prototypical verbs. Therefore, the perfective marker *-le* with Achievement verbs, the progressive marker *zai-* with Activity verbs, and the durative marker *-zhe* with Activity and State verbs are prototypical combinations. Put differently, certain combinations of aspect markers and verbs, although they are grammatical, remain the non-prototypically obvious options. Specifically, the perfective marker *-le* with Accomplishment and Activity verbs and the progressive marker *zai-* with Accomplishment verbs are non-prototypical combinations.

Li and Bowerman (1998) examined the comprehension of Mandarin aspect markers by four- to six-year-old TD children with a sentence-picture matching task. Their results showed that as: First, TD children in all the three age groups (i.e., four-year-old group, five-year-old group, and six-year-old group) obtained higher accuracy when the aspect marker *zai-* was combined with Activity and Semelfactive verbs than with Accomplishment verbs, and when the aspect marker *-le* was combined with Accomplishment verbs than with Activity and Semelfactive verbs. Second, the comprehension of aspect markers increased steadily with children’s age. Six-year-old children were significantly more accurate than four-year-old children for *zai-* (90 and 73.5% for Activity verbs; 73 and 57.5% for Accomplishment verbs) and *-le* (59 and 44.5% for Activity verbs; 80 and 62% for Accomplishment verbs), while *-zhe* did not display clear development across the age range (89 and 89% for Activity verbs; 87 and 78% for State verbs). Third, all three groups of TD children were more accurate on the comprehension of imperfective aspect (*zai-* and -*zhe*; four-year-old group: 69.29%, five-year-old group: 75%, and six-year-old group: 81.43%) than perfective aspect (*-le*; four-year-old group: 55.83%, five-year-old group: 64.5%, and six-year-old group: 72%).^3^

### Acquisition of Mandarin Aspect Markers by Children With DLD

The production of aspect markers by children with DLD acquiring Mandarin shows some similarities to the patterns observed in TD children, in that the production of grammatical aspect is influenced by lexical aspect (Cheung, 2005; He et al., 2013; Chen et al., 2021). In children with DLD, the proportion of imperfective aspect produced (*zai-* and *-zhe*; 45.88%) is also much higher than that of perfective aspect (*-le* and *-guo*; 19.12%; Chen et al., 2021 ^4^), although aspectual production is not necessarily explained by general language abilities and thus may be an area specifically affected in DLD. In addition, among the different aspectual markers examined, while Mandarin-speaking children with DLD reportedly perform similarly to age-matched TD children on producing the pre-verbal marker *zai*-, they perform worse than their TD peers on producing the post-verbal markers *-zhe*, -*le*, and *-guo* (He et al., 2013; Chen et al., 2021). The better mastery of aspect when expressed pre-verbally is captured by the Derivational Complexity Metric (DCM; Jakubowicz, 2005), which postulates that structures involving movement are more complex than those without movement. Indeed, syntactic movement seems to require more computational resources, such as working memory, explaining why movement structures are expected to be particularly challenging for children with DLD, who show difficulties in this realm (Jakubowicz, 2011; Torrens and Yagüe, 2018; Stanford et al., 2019). More specifically for Aspect in Mandarin, given that the aspect projection (AspP) is hierarchically located above the verb projection (VP; see Cinque, 1999), post-verbal (or suffixal) aspect markers in Mandarin would arguably be derived by verb movement (e.g., Roberts, 2019: 382; Huang et al., 2009: 104), in contrast to pre-verbal *zai-*. Hence according to the DCM, *zai-* would be the easiest as has indeed been reported by some authors exploring the production of aspect by children with DLD (e.g., He et al., 2013; Chen et al., 2021). No previous work has examined whether the prediction of DCM also appeared in the comprehension of Mandarin aspect markers, however, which is one of the aims of this study.

There are only two studies that have investigated the comprehension of Mandarin aspect markers by children with DLD. The first study, Cheung (2005) examined the comprehension of Mandarin aspect markers *zai-*, *-le*, and *-guo* by six-year-old children with DLD and found that they performed significantly worse than TD children. However, as the task used in this study only contained four test items and only the mean score of the three aspect markers was provided, it is not clear whether children with DLD performed poorly on perfective aspect or imperfective aspect and whether lexical aspect influenced the comprehension of grammatical aspect. The second study, Yu et al. (2019) investigated the influence of lexical aspect on grammatical aspect on the process of comprehending sentences by seven- to ten-year-old children with DLD as compared to age-matched TD children and language-matched TD children through a self-paced reading task. By measuring the reaction time of *-le* and *-zhe* that followed Activity or Accomplishment verbs, they revealed that children with DLD, like language-matched TD children, processed sentences faster when aspect markers were combined with their prototypical verbs than with non-prototypical verbs. The authors thus proposed that lexical aspect still affected the processing of grammatical aspect in seven- to ten-year-old children with DLD, as they were at an earlier stage of grammatical development.

Other studies on children with developmental disorders, namely, those with autism spectrum disorder (hereafter children with ASD), have also highlighted that the acquisition of Mandarin aspect markers may be problematic. However, while children with ASD display difficulties in producing Mandarin aspect markers (Zhou et al., 2015; Su et al., 2018; Xie and Su, 2018), they appear to fare better in comprehending these same aspect markers (Su and Naigles, 2021). Thus, problems in one modality do not necessarily carry over to the other.

In sum, various studies on Mandarin, a language with overt aspect markers in the absence of overt tense markers, indicate difficulties in the production of aspect in children with DLD; however, work is sparse on comprehension. The current study focuses on the comprehension of the Mandarin aspect markers *zai-*, -*zhe*, and *-le* by children with DLD aged four to six year old as compared to age and nonverbal intelligence matched TD children in the aim of answering three research questions, provided in the following section.

## Research Questions and Predictions

First, do children with DLD show asymmetrical performance on the comprehension of perfective aspect and imperfective aspect in Mandarin, as already revealed by studies that have examined children with DLD who speak English, Greek, and Turkish? Based on the findings of studies on children with DLD who speak other languages and the interaction between tense and aspect, we expect that Mandarin-speaking children with DLD may perform differently depending on whether the marker expresses perfective or imperfective aspect.

Second, do children with DLD show relative difficulty in the comprehension of aspect depending on whether the aspect marker is pre- or post-verbal, as predicted by the DCM and reported for the production of these aspect markers? Mandarin-speaking children with DLD have indeed been shown to struggle in producing the post-verbal markers *-le* and *-zhe*, while they perform as well as TD children on producing the pre-verbal marker *zai-* (He et al., 2013; Chen et al., 2021). We may thus find a similar pattern of performance on the comprehension of aspect markers, namely, children with DLD would show difficulty in comprehending the two post-verbal markers *-le* and *-zhe* but not in comprehending the pre-verbal marker *zai-*.

Third, could the Aspect Hypothesis, which has been shown to predict the performance of TD children on comprehending the three Mandarin aspect markers examined here (Li and Bowerman, 1998), also predict the performance of our participants with DLD? Since seven- to ten-year-old children with DLD are reported to be sensitive to the interaction between lexical aspect and grammatical aspect (Yu et al., 2019), we may expect the preschool children with DLD in this study to show a similar pattern of performance on comprehending Mandarin aspect markers that is fundamentally similar to language-matched TD children as reported by previous studies (e.g., Li and Bowerman, 1998). This pattern of performance on aspect markers *zai-*, *-zhe*, and *-le* attested in children with DLD, if delayed, may also show weaknesses compared to the age and nonverbal intelligence matched TD children of the current study.

## Materials and Methods

### Participants

Twenty-eight four- to six-year-old children participated in this study, 14 children with DLD, and 14 TD controls. Children with DLD were recruited from special education schools, kindergartens, and hospitals while TD controls were recruited from kindergartens. The DLD and TD groups were matched on age and nonverbal intelligence.

Participants’ nonverbal intelligence was measured with the fourth edition of Wechsler Preschool and Primary Scale of Intelligence (WPPSI-IV; Li and Zhu, 2014). Scores of the nonverbal index in the WPPSI-IV test were higher than 85 for all participants. Participants’ general language abilities were tested with the Peabody Picture Vocabulary Test-Revised Chinese Version (PPVT-R; Sang and Miao, 1990) and the Rating Scale for Preschool Children with Language Disorder-Revised Chinese Version^5^ (RSPCLD-R; Lin, 2008). Three scores were retrieved from the language tests, including the score from the PPVT-R, scores of language comprehension and language production from the RSPCLD-R. At least two out of the three scores of children with DLD were 1.25 standard deviations (hereafter, SD) below the norms of their age (following Tomblin et al., 1997), while all the scores of TD children were equal or above norms of their age. Furthermore, parents, teachers, or therapists were interviewed to make sure that no participants had hearing loss, neurological or psychiatric disorders, behavioral disorders, emotional abnormality, etc. Finally, all parents signed consent forms for their children’s participation, which was approved by the Medical Ethics Committee of Xi’an TCM Hospital of Encephalopathy. The descriptive characteristics information of participants is shown in Table 1.

**Table 1:** The participants’ descriptive characteristics.

Group	N	MoA (SD) (Range)	PPVT (SD) (Range)	LC (SD) (Range)	LP (SD) (Range)	NVI (SD) (Range)
DLD	14	61.11 (5.96) (52.93–71.01)	34.57 (14.5) (19–63)	18.57 (4.69) (10–24)	26.64 (5.58) (18–34)	97.5 (5.76) (88–108)
TD	14	63.42 (4.5) (56.52–70.55)	79.29 (16.21) (48–111)	33 (2.11) (29–37)	41.71 (1.73) (39–45)	101.14 (5.74) (91–107)

N, Number of Participants; MoA, Months of Age; LC, Language Comprehension; LP, Language Production, and NVI, the score of the nonverbal index in WPPSI-IV.

Results of independent-sample *t*-tests showed that the DLD and TD groups were matched in age [*t*(26) = 1.153, *p* = 0.259] and nonverbal intelligence [*t*(26) = 1.677, *p* = 0.106]. The TD group, however, scored significantly higher on the general language abilities tests than the DLD group, *t*_ppvt_(26) = 7.693, *p* < 0.001; *t*_LC_(26) = 10.504, *p* < 0.001; *t*_LP_ = 9.649, *p* < 0.001.

### The Sentence-Picture Matching Task

In a sentence-picture matching task, the experimenter articulates a sentence that contains a target grammar point first, and then, the participant needs to choose the picture that matches with the experimenter’s sentence from several given pictures. The experimental materials, procedure, and scoring of the sentence-picture matching task of this study are explained in detail below.

### Materials

The sentence-picture matching task administered included 21 test items and a practice item. Grammatical combinations of the aspect markers with verbs^6^ were used in this task to test whether lexical aspect would have an impact on the comprehension of Mandarin aspect markers. Table 2 shows the numbers of test items (for the full list of the test sentences included in this study, see Supplementary Material). Accomplishment verbs in this study are all verb constellations such as *chi yige pingguo* “eat an apple” and *gai yizuo fangzi* “build a house.” Achievement verbs are all Resultative Verb Compounds that have the features [+dynamic][-durative] [+telic], e.g., *dapo* “break” and *shuaisui* “smash to pieces.” The “*zai-* + Activity verbs” combination is used for the practice item. The target sentences for *zai-*, *-le*, and the “-*zhe* + Activity verbs” combination are with the structure “subject + (*zai*-) + verb+ (*-le/-zhe*) + object” while the target sentences for the “State verbs+ *-zhe*” combination are with the structure “location + verb + -*zhe* + subject.” One verb or verb constellation can be used only once for each “aspect marker + verb” combination.

**Table 2:** Test items in the sentence-picture matching task.

Aspect marker	Total	Activity	Accomplishment	Achievement	State
*zai-*	6	3^*^	3	–	–
*-le*	9	3	3	3^*^	–
*-zhe*	6	3^*^	–	–	3^*^

* *Refers to the prototypical verbs for each aspect marker*.

**Figure 1:**
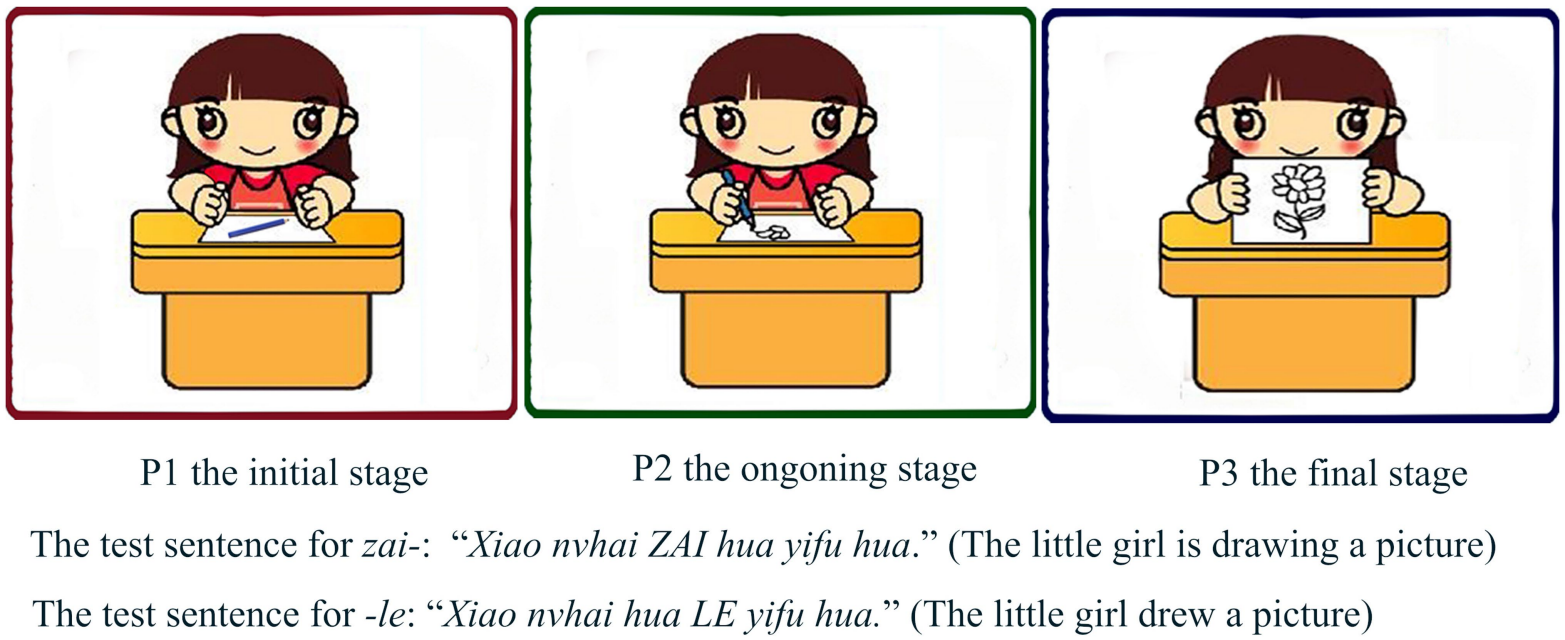
Example for the aspect markers *zai*- and -*le*.

**Figure 2:**
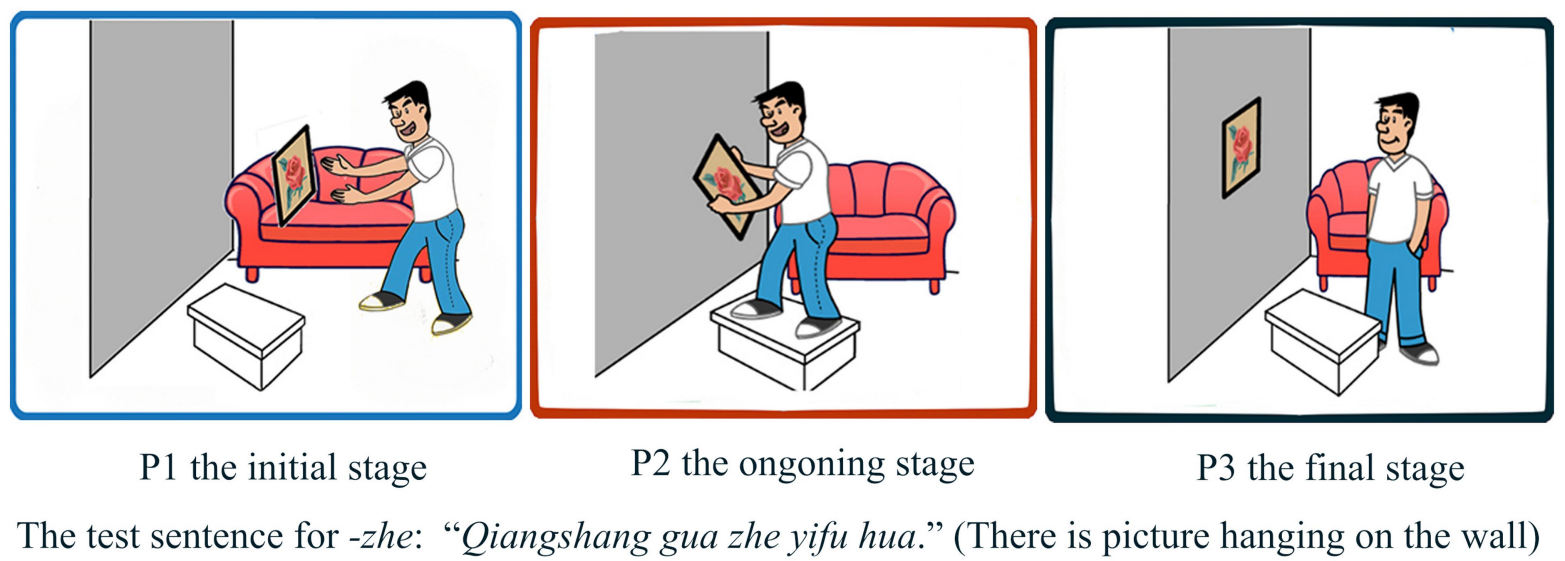
Example for the aspect marker -*zhe*.

Aspect markers *zai-*, *-le*, and -*zhe* are used to indicating the aspectual meaning of a specific event (Smith, 1994). Since a specific event has the initial, ongoing, and final stages (the final stage can refer to either termination or completion of an event), there are three pictures for each test item in the task that represents the initial, ongoing, and final stages of an event, and treated as P1, P2, and P3 from the left to the right. All pictures are cartoon pictures set against a white background, and figures in the pictures are easy to recognize, as shown in Figures 1, 2.

The aspect marker *zai-* represents the ongoing or progressive stage of an event, so the target picture for *zai-* as in the test sentence “*Xiao nvhai ZAI hua yifu hua*.” (The little girl is drawing a picture) is P2 in Figure 1. The aspect marker *-le* indicates the completion or arbitrary termination of an event; thus, P3 in Figure 1 is the target picture for *-le* as in the test sentence “*Xiao nvhai hua LE yifu hua*.” (The little girl drew a picture). Since the basic meaning of the aspect marker *-zhe* is a resultative state that focuses on a stage after the final point of a telic event presented by the verb constellation (Smith, 1994), the target picture for *-zhe* in the test sentence “*Qiangshang gua ZHE yifu hua*.” (There is a picture hanging on the wall) is P3 in Figure 2.

### Procedure

Participants were tested individually in a quiet room. The test items were presented in a pseudo-random order in which adjacent test items represented different aspect markers. There was a filler item between every two to three test items.

At the beginning of the task, which lasted a total of 10 min, an experimenter explained the task to each participant. Then, the practice item was played and feedback was given to the participants to familiarize them with the task procedure. Specifically, the experimenter asked a question followed by an instruction to point in Mandarin, “*Which picture is ‘The girl is drinking a cup of orange juice?’ Please point to it*.” The experimenter praised participants for this practice trial when they pointed to the target picture and corrected them when they pointed to other pictures by saying “*That is not correct, the girl wants to drink a cup of juice in this picture (Pointing to P1), she is drinking (zai-) juice in this picture (Pointing to P2), and she has drunk (-le) the juice up in this picture (Pointing to P3)*. *I asked you to point to the picture in which ‘The girl is drinking a cup of juice*.’ *So you should point to P2*.” For the filler item, the participants were required to choose one object that was articulated by the experimenter, “*Which one is a desk? Please point to it*.” For each test item and filler item, the experimenter displayed the pictures on a screen (laptop or iPad) and gave instructions similar to that in the practice item but without any corrective feedback. The pilot study with 10 three-year-old TD children confirmed that they could understand the instructions of the task. The picture numbers selected by the participants were written down by an assistant.

### Scoring and Data Analysis

Correct answers corresponded to a participant pointing to the target picture (even if the participant corrected the first choice by selecting the correct image quickly afterward), and incorrect answers were the result of pointing to the wrong picture, or giving no response. Then, the data of the DLD and TD groups were analyzed from six aspects: (1) accuracy of perfective aspect versus imperfective aspect; (2) accuracy for each aspect marker; (3) accuracy for each combination of aspect marker with verbs; (4) proportions of non-target pictures chosen by the participants; (5) individual performances of each participant; and (6) correlations between participants’ general language abilities and their performances.

## Results

### Accuracy for Perfective Aspect (*-le*) and Imperfective Aspect (*zai-* and *-zhe*)

The mean, SD, and range of the accuracy for perfective aspect and imperfective aspect are displayed in Table 3. The accuracy scores of *-le* represent the performance of perfective aspect, and the composite scores of *zai-* and *-zhe* represent the performance of imperfective aspect, so as to investigate whether there was an asymmetrical performance on comprehending perfective aspect and imperfective aspect by children with DLD and TD children.

**Table 3:** Accuracy score (%) for perfective aspect and imperfective aspect.

	DLD group			TD group			Mann–Whitney U Test (U)
	Mean	SD	Range	Mean	SD	Range	
*Perfective*	75.4	9.92	56–89	88.10	14.76	56–100	43^*^
*Imperfective*	86.18	7.00	75–100	96.96	4.227	92–100	22^***^
Wilcoxon Test (Z)	2.547^*^			2.099^*^			

^*^Refers to *p* < 0.05, and ^***^refers to *p* < 0.001.

Non-parametric tests were conducted because the data were non-normally distributed (confirmed with Kolmogorov–Smirnov tests). The results of Mann–Whitney U tests showed that the DLD group performed significantly worse than the TD group on both perfective aspect (*p* = 0.011) and imperfective aspect (*p* < 0.001). The Wilcoxon tests revealed that both the DLD and TD groups exhibited significantly better performance on imperfective aspect than on the perfective aspect (*p*_DLD_ = 0.011; *p*_TD_ = 0.036).

### Accuracy for Each Aspect Marker

While children in both the DLD and TD groups were significantly more accurate on imperfective than on perfective aspect, this analysis implied grouping the imperfective markers *zai-* and *-zhe* together. Thus, it remained unclear whether children performed well on both of these markers or only one of them. As a result, we also examined the comprehension of each of the three aspect markers by children in the two groups. The mean, SD, and range of the accuracy for each aspect marker are shown in Table 4.

**Table 4:** Accuracy score (%) for each aspect marker.

		DLD group		TD group			Mann–Whitney U Test (U)
	Mean	SD	Range	Mean	SD	Range	
*zai-*	77.38	14.03	50–100	95.24	7.82	83–100	27^***^
*-le*	75.4	9.92	56–89	88.10	14.76	56–100	43^*^
*-zhe*	95.24	7.82	83–100	98.81	4.46	83–100	77
Friedman Test (Chi^2^)		17.918^***^		6.727^*^			

* Refers to *p* < 0.05, and ^***^refers to *p* < 0.001.

Since the data were non-normally distributed (confirmed with Kolmogorov–Smirnov tests), non-parametric tests were conducted. As shown in Table 4, the results of Mann–Whitney U tests showed that the DLD group was significantly less accurate than the TD group for the *zai-* (*p* = 0.001) and *-le* (*p* = 0.011). On -*zhe*, however, the DLD group performed as well as the TD group (*p* = 0.352).

The results of Friedman tests showed that there were significant differences between the three aspect markers for both the DLD and TD groups. Pairwise comparisons showed that the DLD group obtained significantly higher accuracy for *-zhe* than for *-le* (*p* < 0.001) and *zai-* (*p* = 0.024) while there was no significant difference between *zai-* and *-le*. The TD group was also significantly more accurate for *-zhe* than *-le* (*p* = 0.047), while there was no significant difference neither between *-zhe* and *zai-* nor between *-le* and *zai-*.

To sum up, the inter-group analysis showed that the DLD group got significantly lower accuracy for *zai-* and *-le* than the TD group, while they performed similarly to the TD group on *-zhe*. The intra-group analysis revealed that the DLD group was significantly more accurate for *-zhe* than for *zai-* and *-le*; while the accuracy for *-zhe* was significantly higher than *-le* for the TD group.

### Accuracy for Combinations of Aspect Marker and Verbs

**Figure 3:**
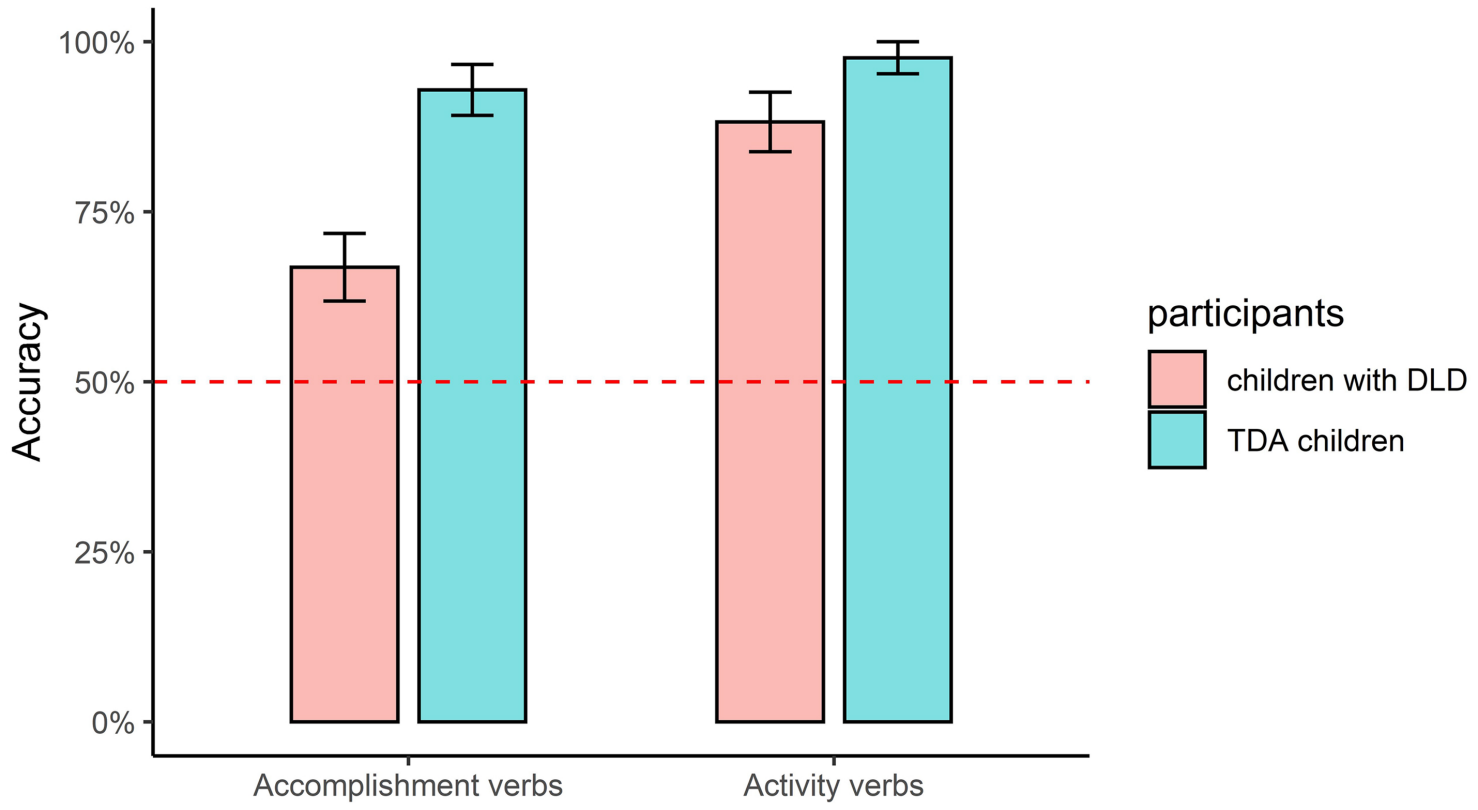
Accuracy of *zai*- being combined with different types of verbs.

**Figure 4:**
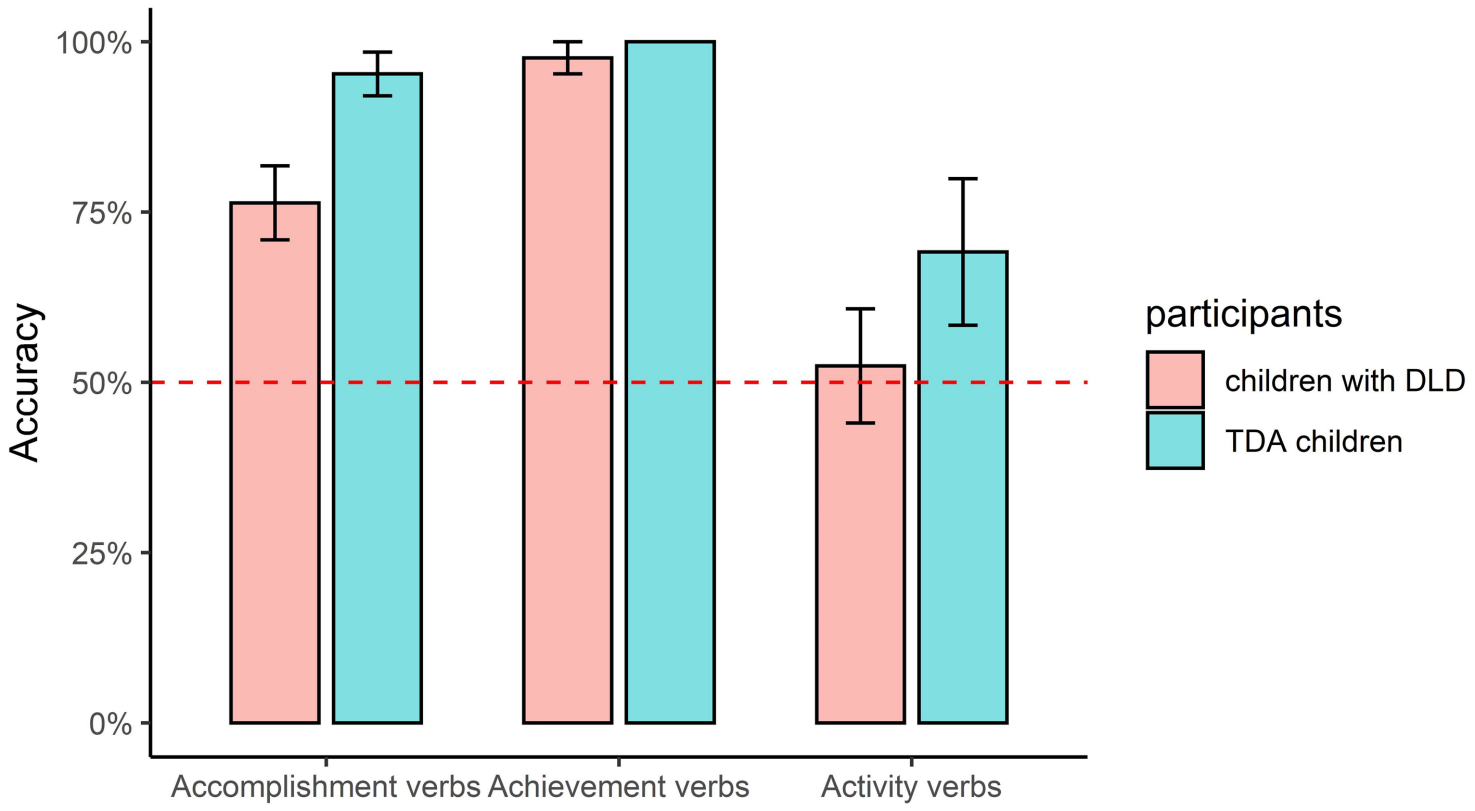
Accuracy of -*le* being combined with different types of verbs.

**Figure 5:**
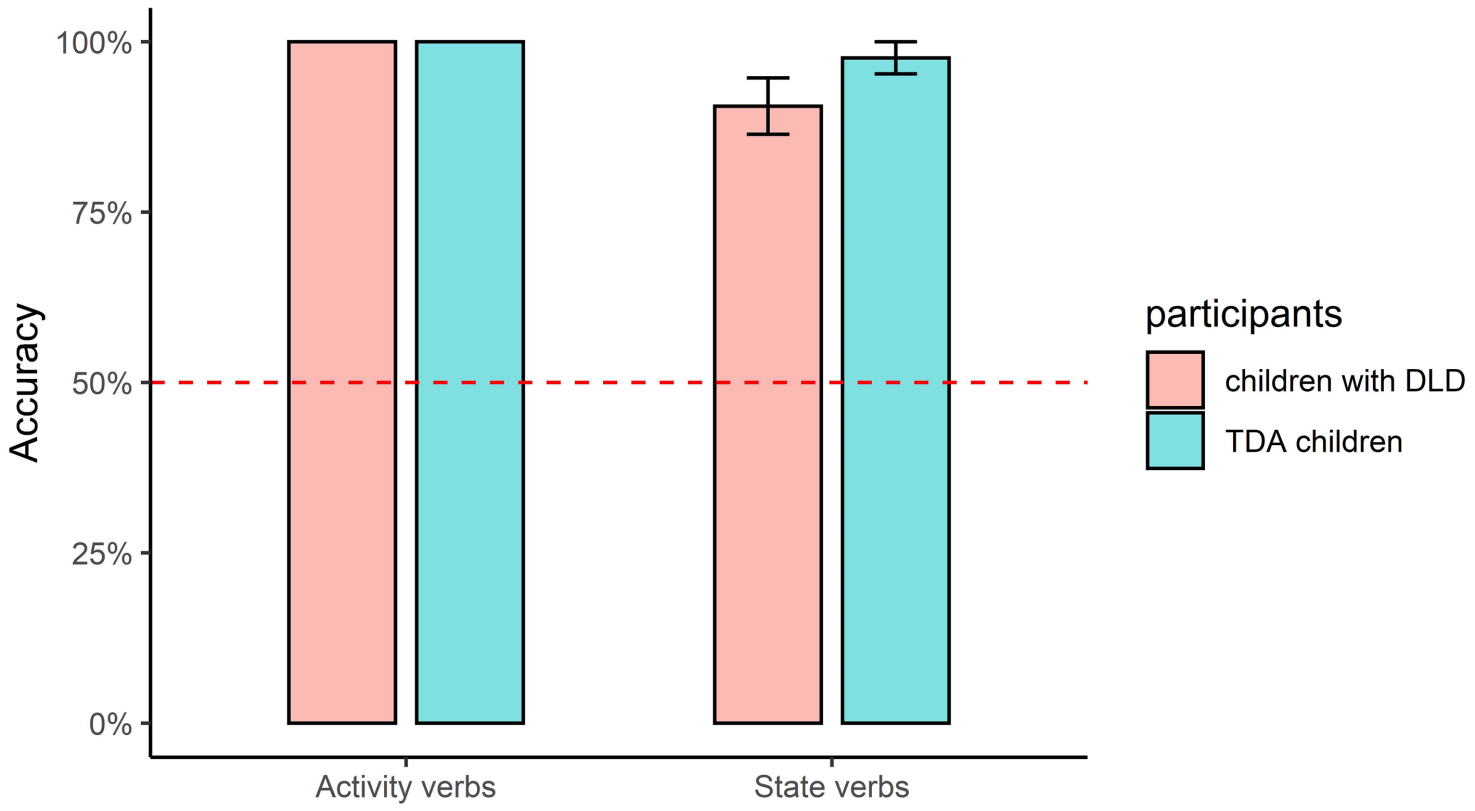
Accuracy of -*zhe* being combined with different types of verbs.

The mean accuracies for the combinations of aspect markers and verbs are shown in Figures 3–5. The figures in this section were produced using the package tidyverse (Wickham et al., 2019) in R (R Core Team, 2020). Non-parametric tests were conducted in this section because the data were non-normally distributed (confirmed with Kolmogorov–Smirnov tests), and the *value of p* represented the adjusted significance for pairwise comparison.

For the progressive marker *zai-* (as shown in Figure 3), Mann–Whitney U tests demonstrated that the DLD and TD groups performed similarly on the “*zai-* + Activity verbs” combination (U = 70, *p* = 0.21); however, the DLD group obtained significantly lower accuracy than the TD group on the “*zai*- + Accomplishment” combination (U = 32, *p* = 0.002). The Wilcoxon tests showed that the DLD group scored significantly higher on the “*zai*- + Activity verbs” combination than the “*zai*- + Accomplishment verbs” combination (*Z* = 2.653, *p* = 0.008), while no significant difference was observed between the two combinations for the TD group (*Z* = 1.00, *p* = 0.317).

For the perfective marker *-le* (as shown in Figure 4), Mann–Whitney U tests showed that the DLD and TD groups performed similarly on the “Achievement verbs+ *-le*” combination (U = 91, *p* = 0.769) and the “Activity verbs+ *-le*” combination (*U* = 66, *p* = 0.15); however, the DLD group was significantly less accurate than the TD group on the “Accomplishment verbs+ *-le*” combination (*U* = 48, *p* = 0.021). Friedman tests demonstrated that there was a significant difference between the combinations of the three types of verbs with -*le* for the DLD group, Chi^2^ = 14.913, *p* = 0.001. The pairwise comparison showed that the accuracy for the “Achievement verbs+ *-le*” combination was significantly higher than the “Activity verbs+ *-le*” combination (*p* = 0.001). A significant difference was also observed between the combinations for the TD group, Chi^2^ = 10.64, *p* = 0.005. However, the pairwise comparisons did not show any significant difference between the three combinations.

For the durative marker *-zhe* (as shown in Figure 5), Mann–Whitney U tests showed that the DLD and TD groups performed similarly on both the “Activity verbs+ -*zhe*” combination (U = 98, *p* = 1.0) and the “State verbs+ *-zhe*” combination (U = 77, *p* = 0.352). Wilcoxon tests showed that the DLD group obtained significantly higher accuracy on the “Activity verbs+ -*zhe*” combination than on the “State verbs + *-zhe*” (*Z* = 2.00, *p* = 0.046), while there was no significant difference between the combinations for the TD group (*Z* = 1.00, *p* = 0.317).

**Figure 6:**
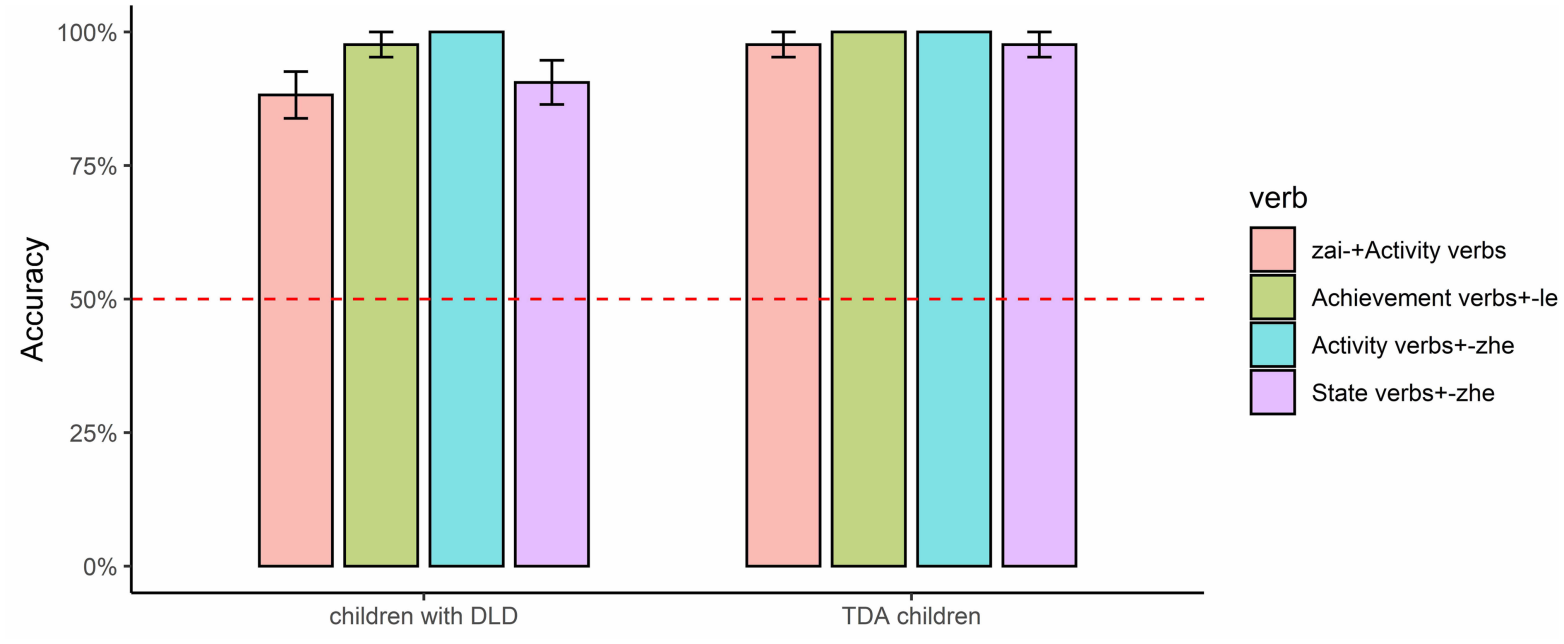
Accuracies for the prototypical combinations of aspect markers with verbs.

Finally, the prototypical combinations between the aspect markers and verbs (as shown in Figure 6) within each group were compared.

Friedman tests demonstrated that there was no significant difference between “*zai-* + Activity verbs,” “Achievement verbs+ -*le*,” “Activity verbs+ -*zhe*,” and “State verbs+ -*zhe*” for the DLD group, Chi^2^ = 7.286, *p* = 0.063; the same held for the TD group, Chi^2^ = 2.000, *p* = 0.572.

The aspect marker *zai-* is prototypically combined with Activity verbs, *-le* with Achievement verbs, and *-zhe* with both Activity and State verbs. The analysis demonstrated that children in the DLD group got higher accuracy when *zai-* was combined with Activity verbs than with Accomplishment verbs, when -*le* was combined with Achievement verbs than with Activity verbs, and when *-zhe* was combined with Activity verbs than with State verbs^7^. Furthermore, there were no differences between the four prototypical combinations between aspect markers and verbs (i.e., between “*zai-* + Activity verbs,” “Achievement verbs+ -*le*,” “Activity verbs+ -*zhe*,” and “State verbs+ -*zhe*”) for both the DLD and TD groups. As a result, it can be concluded that children in the DLD group performed better when Mandarin aspect markers were combined with their prototypical verbs than with the non-prototypical verbs. The inter-group analysis showed that the DLD group obtained significantly lower accuracy than the TD group when *zai-* and *-le* were combined with Accomplishment verbs.

### Error Types

Since the three pictures in each test item represent the initial, ongoing, and completed stages of an event, we computed the proportions of the two non-target pictures chosen by participants for *zai-* and *-le*, respectively (*-zhe* was excluded since all participants performed well on it). This allowed us to explore whether there were certain error patterns in choosing pictures by children in the DLD group. The proportions of the two non-target pictures are shown in Table 5.

**Table 5:** Proportions (%) of non-target pictures for aspect markers *zai-* and *-le.*

Aspect marker	Verbs	Non-target picture	DLD group Mean (SD)	TD group Mean (SD)	M–W test (U)
*zai-*	ACT	P1	7.14 (14.19)	0 (0)	77
		P3	4.76 (12.1)	2.38 (8.91)	91
	ACC	P1	2.38 (8.91)	2.38 (8.91)	98
		P3	30.95 (15.82)	4.76 (12.1)	27^***^
*-le*	ACH	P1	0 (0)	0 (0)	98
		P2	2.38 (8.91)	0 (0)	91
	ACC	P1	9.52 (15.63)	0 (0)	70
		P2	14.28 (21.54)	4.76 (12.1)	76
	ACT	P1	11.9 (21.11)	4.76 (12.1)	83
		P2	35.71 (24.34)	26.19 (37.39)	71.5

ACT refers to Activity verbs, ACC refers to Accomplishment verbs, and ACH refers to Achievement verbs; ^***^refers to *p* < 0.001.

From Table 5, it can be seen that children in the DLD group made significantly more mistakes than those in the TD group by choosing P3 (the completed stage of an event) on the “*zai*- + Accomplishment” combination. For other combinations of aspect markers with lexical aspect, there were no significant differences between the two groups. Therefore, it can be proposed that children in the DLD group had similar patterns with those in the TD group in choosing the non-target pictures for *zai-* and *-le*.

Furthermore, it is worth underlining that children in the DLD group chose 30.95% of P3 (the completed stage) on the “*zai-* + Accomplishment verbs” combination and 35.71% of P2 (the ongoing stage) on the “Activity verbs+ -*le*” combination; while, children in the TD group chose 4.76% of P3 (the completed stage) on the “*zai-* + Accomplishment verbs” combination and 26.19% of P2 (the ongoing stage) on the “Activity verbs+ -*le*” combination. This pattern of performance indicates that lexical aspect might also have an impact on young children’s event recognition, especially on those with DLD.

### Individual Performance

The performance of each individual on comprehending the three aspect markers is shown in Table 6.

**Table 6:** Individual performance on each aspect marker.

DLD	*zai-*	*-le*	*-zhe*	TD	*zai-*	*-le*	*-zhe*
1	100%	55.56%	100%	1	100%	100%	100%
2	83.33%	77.78%	83.33%	2	100%	100%	100%
3	83.33%	77.78%	83.33%	3	100%	66.67%	100%
4	83.33%	77.78%	100%	4	100%	66.67%	100%
5	83.33%	77.78%	100%	5	100%	100%	100%
6	83.33%	88.89%	100%	6	100%	88.89%	100%
7	83.33%	77.78%	100%	7	100%	55.56%	83.33%
8	83.33%	66.67%	83.33%	8	100%	88.89%	100%
9	83.33%	88.89%	100%	9	100%	100%	100%
10	83.33%	66.67%	83.33%	10	100%	88.89%	100%
11	66.67%	66.67%	100%	11	83.33%	88.89%	100%
12	66.67%	66.67%	100%	12	83.33%	100%	100%
13	50%	88.89%	100%	13	83.33%	100%	100%
14	50%	77.78%	100%	14	83.33%	88.89%	100%

When we consider the performance of the 14 children in the DLD group, we see that none of them scored 100% on all the three aspect markers, two of them only obtained 50% on *zai-*, and one child received 55.56% on *-le*; meanwhile, two children obtained 66.67% on both *zai-* and *-le* although they got 100% on *-zhe*. In contrast, four children in the TD group got 100% on all the three aspect markers, and only three of them scored less than 70% on *-le*. Therefore, as Table 6 indicates, no child in the DLD group did as well as those in the TD group on *zai-* and -*le* in the sentence-picture matching task, although various children in the DLD group did as well as their TD peers on -*zhe*.

### Correlation Analysis

Since the general language abilities of children with DLD were significantly lower than those of TD children, it is reasonable to hypothesize that the poor performance of children with DLD on comprehending the aspect markers *zai-* and *-le* may be related to their poor general language abilities. Therefore, we conducted Pearson analyses to examine whether there were correlations between the performance of children in the two groups on comprehending *zai-* and *-le* with the three dimensions of their general language abilities^8^, namely, PPVT, language comprehension, and language production. The Arc-sine transformations were applied to the percentage data. The results of correlation analyses are shown in Table 7.

**Table 7:** The result of correlation analysis (r).

	DLD group		TD group	
		*zai-*	*-le*	*zai-*	*-le*
PPVT	−0.019	0.289	−0.110	0.105
LC	0.225	−0.143	−0.078	0.316
LP	−0.136	0.035	−0.108	0.371

*LC, language comprehension in the RSPCLD-R; LP, language production in the RSPCLD-R*.

Table 7 illustrates that there were no significant correlations between the three dimensions of general language abilities of children in the two groups and their performance on comprehending *zai-* and *-le*.

## Discussion

This study investigated the comprehension of three Mandarin aspect markers *zai-*, *-zhe*, and *-le* by preschool Mandarin-speaking children with DLD as compared to age and nonverbal intelligence matched TD children with a sentence-picture matching task. The findings shed light on our three research questions, individually tackled below:

### Do Children With DLD Show Asymmetrical Performance on the Comprehension of Perfective Aspect and Imperfective Aspect in Mandarin?

This study is the first study that compares the performance of Mandarin-speaking children with DLD on the comprehension of perfective aspect (*-le*) and imperfective aspect (*zai*- and *-zhe*). We found that children with DLD indeed showed better performance on comprehending imperfective aspect than perfective aspect when imperfective markers were considered on a whole. The performance of children with DLD also followed a similar pattern to their age and nonverbal intelligence matched TD peers, although they were less accurate than TD children on the comprehension of both perfective aspect and imperfective aspect. The asymmetrical performance of children with DLD between perfective aspect and imperfective aspect was also found in their production. Chen et al. (2021) reported that the proportion of imperfective aspect was much higher than that of perfective aspect in the production of children with DLD.

The findings reported here for comprehension and in Chen et al. (2021) for production follow from van Hout’s Semantic Complexity Hypothesis (van Hout, 2008). According to the Semantic Complexity Hypothesis, the semantics of simple semantic operations are acquired earlier than complex semantic operations. Since imperfective aspect and present tense are naturally conflated linguistic categories (Wagner, 2001), the semantic operations of imperfective aspect are simpler than that of perfective aspect under present tense. Although Mandarin is a tense-less language, the sentence-picture matching task in this study offers a “here and now” context that corresponds to present tense, by displaying three pictures that represent the initial, ongoing, and completed stages of an event. Therefore, according to the Semantic Complexity Hypothesis, processing the imperfective markers *zai-* and *-zhe* would require simpler semantic operations than processing the perfective marker *-le* under present tense in this study.

However, since there are two aspect markers (the progressive marker *zai-* and the durative marker -*zhe*) expressing imperfective aspect in Mandarin, it was important to check that the better performance of children with DLD on comprehending imperfective aspect applies to both aspect markers, rather than being driven by only one. Findings for these individual markers will be discussed in the next section, when we focus on the potential effects of the pre- versus post-verbal realization of these elements.

### Do Children With DLD Show Asymmetrical Performance Depending on Whether the Aspect Marker Is Pre- or Post-verbal?

While we expected children with DLD to perform poorly on comprehending the two post-verbal markers *-le* and *-zhe* and do well on comprehending the pre-verbal marker *zai-*, as already reported for production by He et al. (2013) and Chen et al. (2021), the results of this study rather showed that the DLD group obtained significantly lower accuracy than the TD group on *zai-* and *-le*, while they performed similarly to their TD peers on *-zhe*. The global pattern of performance was also confirmed on the individual level for both the DLD and TD groups. This suggests that the pre- or post-verbal occurrence of aspect markers impacts comprehension differently from how it impacts production, such that comprehension and production modalities might involve different processing mechanisms (see also Grüter, 2005 for similar findings with clitic pronouns in children with DLD).

The performance of children with DLD (mean: 61.11 months old; range: 52.93–71.01) in this study was similar to that reported for four-year-old TD children (mean: 4;2; range: 3;11–4;4) in Li and Bowerman (1998). First, both groups of children obtained lower accuracy for *zai-* and *-le* than other groups [TD group in this study (63.4-month-old) and six-year-old group in the study of Li and Bowerman, 1998] while they did as well as other groups for *-zhe*. Second, similar to the four-year-old group in the study of Li and Bowerman (1998), the DLD group in this study was more likely to be impacted by lexical aspect on comprehending aspect markers than other groups, although the pattern of interaction between aspect markers and lexical aspect was similar across groups.

In sum, these findings illustrate that children with DLD, like their TD peers, performed better on imperfective aspect than on perfective aspect. However, upon closer inspection, it can be observed that the accuracy of the imperfective marker *zai-* was as lower as the perfective marker *-le*. Our results, which contain scores for different imperfective markers, rather suggest that an individual imperfective marker (i.e., *zai-*) may pose more problems than another imperfective marker (i.e., *-zhe*). Thus, a more nuanced view of aspectual development emerges, and an account in terms of the global perfective-imperfective categories of aspect markers cannot account for the fine-grained patterns of comprehension of aspect markers by Mandarin-speaking children with DLD.

### Does the Aspect Hypothesis Predict the Performance of Children With DLD on Comprehending the Three Mandarin Aspect Markers?

In line with the results of Yu et al. (2019), this study also found that the comprehension of aspect markers by children with DLD was affected by lexical aspect. Specifically, in both of our studies, higher accuracy was observed when aspect markers were combined with their prototypical verbs than with non-prototypical verbs. Also like the findings in Yu et al. (2019) showing that seven- to ten-year-old children with DLD performed similarly to younger, language-matched TD peers, the 5 year olds with DLD in the current study showed similar performance to the younger TD children in Li and Bowerman of four-years (1998) on the comprehension of Mandarin aspect markers.

Our inter-group comparisons demonstrated that children in the DLD group were comparable to those in the TD group in terms of accuracy and error patterns, providing the aspect markers were combined with their prototypical verbs. However, those with DLD were less accurate than their TD peers when *zai*- and *-le* were combined with Accomplishment verbs. Similar findings for twenty-year-old Chinese learners of English were also reported by Zeng et al. (2021), who investigated the interaction of lexical aspect and grammatical aspect on the comprehension of English past tense and progressive aspect by Chinese-English learners with a self-paced reading task. Like age-matched native English speakers, these L2 adults performed much better on processing the combinations of tense-aspect markers with prototypical verbs than with non-prototypical verbs, and they performed worse than native English speakers only when tense-aspect markers were combined with non-prototypical verbs.

It is worth noting that children in the DLD group of our study were significantly less accurate than those in the TD group for the “Accomplishment verbs+ - *le*” combination. Previous studies on the use of Mandarin aspect markers have revealed that TD children mostly combine the perfective marker *-le* with Achievement verbs at the early stage of aspectual development. These children only occasionally combine *-le* with Accomplishment verbs as well as Activity verbs initially, although the tendency weakens with aspectual development (Jin and Hendriks, 2005; Chen and Shirai, 2010). A similar finding was reported by Shirai and Andersen (1995) in the acquisition of English regular and irregular past tense, namely, that children used regular or irregular past tense morphology predominantly with Achievement verbs first. The worse performance of children with DLD on the “Accomplishment verbs+ - *le*” combination than their TD peers further supports the hypothesis that four- to six-year-old children with DLD are at the early stage of aspectual development.

Taken together, our study on preschool participants with DLD coupled with others on older children with DLD, as well as L2 learners, all seem to suggest that comprehension of Mandarin aspect markers can be impacted by lexical aspect, in a way that echoes findings for younger TD samples. Therefore, our cohort appears to be at an earlier stage of aspectual development as compared with their age and nonverbal intelligence matched TD peers, when difficulty can still be observed in comprehending aspect markers when they are combined with their non-prototypical verbs, as predicted by the Aspect Hypothesis.

Unlike the finding that lexical aspect affects the comprehension of Mandarin aspect markers by children with DLD in Yu et al. (2019) and this study, lexical aspect does not impact the comprehension of grammatical aspect by English and Spanish-speaking children (and adolescents) with DLD (Leonard and Deevy, 2010; Grinstead et al., 2013; Stuart and van der Lely, 2015). This difference between Mandarin on the one hand and English and Spanish on the other cannot be attributed to the level of aspectual development of the participants since across studies they were all over 7 years old, so their aspectual development should have been mature. The experimental paradigms cannot clearly explain the difference either since the two studies on Mandarin-speaking children with DLD used different paradigms, while one on English, namely, Stuart and van der Lely (2015) used the same sentence-picture matching task as the current study on Mandarin. Instead, these different findings on whether lexical aspect affects the comprehension of grammatical aspect or not might relate to the fact that in Mandarin, where tense is not overtly marked, the interpretation of aspect may rely more intricately on lexical information, while overt tense may contribute to the interpretation of aspect in English and Spanish. Further studies should focus on children with DLD who speak other tense-less languages (such as Burmese, Thai, and Vietnamese) to determine the role of tense marking on the interaction between lexical aspect and grammatical aspect.

Since children with DLD obtained lower accuracy for aspect markers *zai-* and -*le*, it raises the question of whether this poor performance was linked to their general language abilities. However, the correlation analysis identified no significant links between the performance of children with DLD on comprehending *zai-* and *-le* and their general language abilities. These findings are consistent with that of Chen et al. (2021) who investigated the production of Mandarin aspect markers by children with DLD. More specifically, the latter does not reveal any significant correlations between the performance of children with DLD on the production of aspect markers and their general language abilities either. Our study and that of Chen et al. (2021) are moreover consistent with the findings reported by Su and Naigles (2021) for two- to six-year-old children with ASD. These authors also observed no link between the mean length of utterance and vocabulary production scores with performance on comprehending *zai-* and *-le* during the Intermodal Preferential Looking task. Thus, children with language difficulties, be they DLD or ASD, may show challenges in the realm of aspect that are not immediately explained by their global linguistic skills.

The pattern of performance of children with DLD on comprehending aspect markers in this study seems to be the result of a delay in aspectual development which is moreover specifically challenged by non-prototypical combinations of aspect markers and verbs. Furthermore, error patterns of children in the DLD group also showed that they were impacted by lexical aspect on event recognition. Namely, among the test items for the “*zai-* + Accomplishment verbs” combination, children with DLD made 30.95% choice of the third picture (the completed stage of an event) and made 35.71% choice of the second picture (the ongoing stage of an event) for the “Activity verbs+ *- le*” combination.

Finally, the comparison between perfective aspect and imperfective aspect here also suggests that preschool children with DLD are not deviant in their grammatical development, because they followed a similar comprehension pattern to their age and nonverbal intelligence matched TD peers, namely, imperfective aspect being easier than perfective aspect. Moreover, their poor performance on the comprehension of aspect was nonetheless similar to younger TD children (of around 4 years) in Li and Bowerman (1998), suggesting that they display a delayed pattern of aspectual development.

Since the ability of children with DLD on the comprehension of Mandarin aspect markers might be delayed (inferred from their similar performance with younger, four-year-old TD children in the study of Li and Bowerman (1998)), it is reasonable to propose that training on the use of aspect markers could benefit Mandarin-speaking children with DLD since it has been previously shown for interventions addressing past tense for English-speaking children with DLD (Leonard et al., 2006; Garraffa et al., 2018; Owen Van Horne et al., 2018; Calder et al., 2021). Garraffa et al. (2018) showed that children with DLD can capitalize on explicit training to overcome their syntactic challenges. Calder et al. (2021) reported that by increasing the learner’s awareness of the goal of intervention, past tense production in early school-age children with DLD was improved. Furthermore, Owen Van Horne et al. (2018) revealed that treatment outcomes on the use of regular past tense *-ed* by English-speaking children with DLD were influenced by verbs’ lexical, aspectual, and phonological properties, and “hard” verbs (i.e., verbs that are atelic, lower-frequency, and phonologically complex) lead to greater gains. Therefore, offering explicit intervention and selecting the combinations of aspect markers with their non-prototypical verbs might be especially beneficial to incorporate in training protocols addressing aspect markers in children with DLD.

### Limitations

This work presents various limitations, two of which are acknowledged here.

First, language-matched TD children were not included. Although the findings of other studies on Mandarin-speaking TD children, Chinese-English learners, and children with DLD who speak English, Greek, Spanish, and Turkish provide indirect evidence that preschool children with DLD in our study were delayed rather than deviant in aspectual development, future studies including younger, language-matched TD children remain necessary to confirm this conclusion.

Second, there was only one practice item and it contained the “*zai-* + Activity verbs” combination. That children were already exposed to this combination during the warm-up might have facilitated their comprehension of *zai-* during the test items. Nonetheless, the results showed that children with DLD performed better on *-zhe* than on *zai-*. Even so, the practice item that familiarizes the task procedure should be fundamentally different from the test items in future studies.

## Conclusion

This study investigated the comprehension of three typical Mandarin aspect markers *zai-*, *-zhe*, and *-le* by four- to six-year-old Mandarin-speaking children with DLD as compared to age and nonverbal intelligence matched TD children through a sentence-picture matching task. Results showed that children with DLD performed worse than their TD peers on the comprehension of both perfective aspect and imperfective aspect although they followed a similar pattern to TD children, namely, that imperfective markers grouped together yielded higher accuracy than perfective aspect. Still, when specific aspect markers were considered, children with DLD obtained significantly lower accuracy than TD children on one imperfective *zai-*, like they did on perfective *-le*, while they performed similarly to TD children on imperfective *-zhe*. Thus, the imperfective/perfective dichotomy cannot account for the more nuanced pattern observed here. Moreover, performance was affected by lexical aspect, in that children with DLD obtained higher accuracy when *zai-* and *-le* were combined with their prototypical verbs than with their non-prototypical verbs, at a level reminiscent of that previously reported for younger TD peers. These findings suggest that children with DLD might be at an earlier stage of aspectual development as compared to age and nonverbal matched TD children. Given this delay, training protocols addressing the use of aspect markers may be appropriate for children with DLD.

## Data Availability Statement

The raw data supporting the conclusions of this article will be made available by the authors upon request, without undue reservation.

## Ethics Statement

This study involving human participants were reviewed and approved by the Medical Ethics Committee of Xi’an TCM Hospital of Encephalopathy. Written informed consent to participate in this study was provided by the participants’ legal guardian/next of kin.

## Author Contributions

Both authors listed have made a substantial, direct, and intellectual contribution to the work and approved it for publication.

## Funding

The Swiss National Science Foundation (grant no. PR00P1_193104/1), the National Social Science Foundation of China (grant no.17AYY08), and the China Scholarship Council.

## Conflict of Interest

The authors declare that the research was conducted in the absence of any commercial or financial relationships that could be construed as a potential conflict of interest.

## Publisher’s Note

All claims expressed in this article are solely those of the authors and do not necessarily represent those of their affiliated organizations, or those of the publisher, the editors and the reviewers. Any product that may be evaluated in this article, or claim that may be made by its manufacturer, is not guaranteed or endorsed by the publisher.

## Footnotes

^1^Zhang and Li (2021) argue that *zai-* is independent tense marker when it is used alone. However, if it suffices for a marker to be able to occur on its own for this marker to be a tense marker, then the particles -*zhe* and *-le* could also be tense markers, which even they do not claim. In addition, the authors argue that *zai-* is a tense marker expressing present tense in the following sentence “*Diqiu zai rao taiyang zhuan*” (The earth revolves around the sun). However, this very sentence is better expressed with the particle *-zhe* as in “*Diqiu rao zhe taiyang zhuan,*” where *zai-* is not present and yet present tense is encoded. Finally, various so-called ungrammatical sentences they provide are perfectly acceptable sentences for native Mandarin speakers, e.g., “**wo zhu zai lutedan*” (I lived in Rotterdam). Therefore, we continue to adopt the traditional viewpoint rather than Zhang and Li’s in this study.

^2^Smith (1997: 75) regards the resultative state as marked imperfective aspect since it focuses on the interval following the final point of a telic event.

^3^The data are obtained from Table 1 in Li and Bowerman (1998: 325). The authors did not include Achievement verbs in their study in that their study was designed to include perfective-imperfective pairs with one verb that could be combined both with *-le* and *zai-* or *-le* and *-zhe* but Achievement verbs cannot be combined with the progressive marker *zai-* or the durative marker *-zhe*. Here, the data for Activity verbs (i.e., atelic verbs) with *zai-* and *-le* are the mean of percentage data for Activity verbs and Semelfactive verbs (the two types of verbs behave similarly in their combinations with aspect markers), and that for Accomplishment verbs (i.e., telic verbs) are the mean of percentage data for Resultative Accomplishment verbs and Locative Accomplishment verbs; the data for perfective aspect are the mean of six percentage data for *-le*; and the data for imperfective aspect are the mean of five percentage data for *zai-* and two percentage data for *-zhe*.

^4^The data are taken from Table 4 in Chen et al. (2021: 199).

^5^The detailed description of the PPVT-R and the RSPCLD-R is shown in Supplementary Material.

^6^Semelfactive verbs were not included in the task because this type of verbs is same to Activity verbs in the combination with aspect markers.

^7^Although children in the DLD group got lower accuracy on the “State verbs+ -*zhe*” combination than the “Activity verbs+ *-zhe*” combination, the mean accuracy on the “State verbs+ -*zhe*” was 90.48%; thus, it was very high.

^8^The same results of Pearson analysis were obtained for each group with the composite scores of PPVT, language comprehension, and language production.
