# Supplementary material for: Comprehension of Mandarin Aspect Markers by Preschool Children With and Without Developmental Language Disorder
Source: Front Psychol. 2022 Apr 28;13:839951. doi: 10.3389/fpsyg.2022.839951 (PMC9097452; doi:10.3389/fpsyg.2022.839951)
Supplement: Supplementary file 1 [file Data_Sheet_1.pdf]

# Appendix A

**1. PPVT-R**

The Peabody Picture Vocabulary Test-Revised (PPVT-R) is used to measure an individual's receptive vocabulary for Mandarin Chinese and provides a quick estimate of verbal ability. The PPVT-R consists of 175 stimulus words and 175 corresponding images. Each image contains 4 black-and-white drawings, one of which best represents the meaning of the corresponding stimulus word. There are also 5 training words presented with corresponding images. Modifications from the English version of PPVT-R in the creation of the Chinese version include 15 stimuli (8.57% of total vocabulary), and 15 drawings (2.14% of the total pictures), so as to render this version more easily recognizable by Chinese children. For the Chinese version of PPVT-R, the internal consistency is .99; the test-retest reliability is .938. The norms based on the raw score of PPVT-R are provided for three- to nine-year-old children by Sang and Miu (1994) based on 600 children who come from Shanghai.

**2. RSPCLD-R**

The Rating Scale for Pre-school Children with Language Disorder-Revised Chinese Version (RSPCLD-R) is a tool used to evaluate the language comprehension, language production, expressive vocabulary, pronunciation, and speech fluency of children who are between 3;0 years old and 5;11 years old. It aims to screen out the children who are delayed or impaired in language. There are four sub-tests in the RSPCLD-R. The five test items in the first subtest are used to make the participant feel relaxed and to evaluate children’s speech fluency with question-answering. The second sub-test includes 29 test items, which are used to evaluate children’s language comprehension ability with picture-choice, question-answering, and story quizzes. The third sub-test assesses children’s expressive vocabulary and pronunciation manner with 13 test items by asking children to speak out the names of objects in the picture cards. There are 18 test items evaluating children’s language production ability in the fourth sub-test with question-answering, sentence repetition, sentence-making and story-telling. The scores of the second and fourth sub-tests are used to screen children with DLD in this study. The internal consistency for the comprehension part is .91, and the production part is .94; the test-retest reliability is .93 for the comprehension part and .92 for the production part; the size of the norm was 725 children. The data for the norms were collected in Taiwan.

## Appendix B

General instruction: XX. Let's play a game. I will say a sentence, please point to the picture that matches what it means. All right?

**The progressive maker *zai-* +Activity verbs**

1. 哥哥 在 游泳。
Gege zai youyong.
brother ZAI swim
'The brother is swimming.'

2. 哥哥 在 洗手。
Gege zai xishou.
brother ZAI wash hands
'The brother is washing his hands.'

3. 姐姐在 浇花。
Jiejie zai jiaohua.
sister ZAI water flower
'The sister is watering a flower.'

**The progressive maker *zai-* +Accomplishment verbs**

4. 姐姐在 喝 一杯 果汁。
Jiejie zai he yibei guozhi
sister ZAI drink one-CL juice
'The sister is drinking a cup of juice.'

5. 叔叔 在 盖 一座 房子。
Shushu zai gai yizuo fangzi.
uncle ZAI build one-CL house
'The uncle is building a house.'

6. 姐姐 在 吃 一个 苹果。
Jiejei zai chi yige pingguo.
sister ZAI eat one-CL apple
'The sister is eating an apple."

**The perfective marker *-le* +Achievement verbs**

7. 叔叔 摔碎 了 一个 瓶子。
Shushu shuaisui le yige pingzi.
uncle throw-smashed LE one-CL bottle
'The uncle smashed a bottle to pieces.'

8. 小猫 钓到 了 一条 鱼。
Xiaomao diaodao le yitiao yu.
little-cat fish-get LE one-CL fish
'The little cat hooked a fish.'

9. 哥哥 踢破 了 门。
Gege tipo le men.
brother kick-broken LE door
'The brother kicked the door and it smashed.'

**The perfective marker *-le* +Accomplishment verbs**

10. 哥哥 折 了 一个 纸飞机。
Gege zhe le yige zhifeiji.
brother folder LE one-CL paper-plane

‘The brother made a paper plane.’

11. 小猫　吃 了 一条 鱼。

Xiaomao chi le yitiao yu.
little-cat eat LE one-CL fish
‘The litter cat ate a fish.’

12. 姐姐 画 了 一幅　画。

Jiejie hua le yifu hua.
sister draw LE one-CL picture
‘The sister drew a picture.’

**The perfective marker *-le* +Activity verbs**

13. 哥哥 拖 了 地。

Gege tuo le di.
brother mop LE floor
‘The brother mopped the floor.’

14. 姐姐 吃 了 饭。

Jiejie chi le fan.
sister eat LE meal
‘The sister had a meal.’

15. 姐姐 洗　了 手。

Jiejie xi le shou.
sister wash LE hands
‘The sister washed her hands.’

**The durative marker *-zhe* +Activity verbs**

16. 姐姐 牵　着 狗。

Jiejie qian zhe gou.
sister leash ZHE dog
‘The sister is leashing a dog.’

17. 姐姐 戴 着　帽子。

Jiejie dai zhe maozi.
sister wear ZHE hat
‘The sister is wearing a hat.’

18. 姐姐 拿　着　苹果。

Jiejie na zhe pingguo.
sister take ZHE apple
‘The sister is taking an apple.’

**The durative marker *-zhe* +State verbs**

19. 墙上　　挂　着　一幅　画。

Qiangshang gua zhe yifu hua.
wall-on hang ZHE one-CL picture
‘There is a picture hanging on the wall.’

20. 床上　　睡　着　一头　小猪。

Chuangshang shui zhe yitou xiaozhu.
bed-on sleep ZHE one-CL piglet
‘There is a piglet sleeping on the bed.’

21. 椅子上　坐 着 一个　小男孩。

Yizishang zuo zhe yige xiaonanhai.
chair-on sit ZHE one-CL little boy

‘There is a boy sitting on the chair.’
